# Herbal medicine treatment for Alzheimer disease: A protocol for a systematic review and meta-analysis

**DOI:** 10.1097/MD.0000000000021745

**Published:** 2020-08-14

**Authors:** JiEun Lee, Chul Jin, Seung-Yeon Cho, Seong-Uk Park, Woo-Sang Jung, Sang-Kwan Moon, Jung-Mi Park, Chang-Nam Ko, Ki-Ho Cho, Seungwon Kwon

**Affiliations:** a Department of Korean Medicine Cardiology and Neurology, Graduate School; b Department of Cardiology and Neurology, College of Korean Medicine, Kyung Hee University, Seoul, Republic of Korea.

**Keywords:** Alzheimer disease, herbal medicine, meta-analysis, systematic review

## Abstract

**Background:**

Alzheimer disease (AD) is a leading progressive neurodegenerative disease worldwide, but treating it is challenging in clinical practice. This review is aimed at evaluating the efficacy and safety of herbal medicine for treating AD.

**Methods and analysis:**

We will search for randomized controlled trials related to the effect and safety of herbal medicine for AD in the following databases: PubMed, Cochrane Central Register of Controlled Trials, Excerpta Medica Database, China National Knowledge Infrastructure database, Oriental Medicine Advanced Searching Integrated system, Korean Traditional Knowledge Portal, and Citation Information by National Institute for Informatics. The risk of bias will be evaluated using the Cochrane risk-of-bias assessment tool. After screening the studies, a meta-analysis will be performed. The primary outcome will be the Mini-Mental State Examination score. Secondary outcomes will consist of other scales for cognitive function and other aspects, such as behavioral and psychological symptoms and plasma levels of amyloid-β.

**Results:**

This study will provide the current status of evidence for herbal medicine to treat AD.

**Conclusion:**

The results of this review will determine the efficacy and safety of herbal medicine for AD.

**Ethics and dissemination:**

Ethical approval is not required, as this study is based on a review of published research. This review will be published in a peer-reviewed journal and disseminated both electronically and in print.

**Trial registration number:**

Research Registry reviewregistry933.

## 1 Introduction

Alzheimer disease (AD) is a chronic progressive neurodegenerative disorder. It is the leading cause of dementia, accounting for approximately 60% to 70% of the cases. Presently over 47 million people worldwide suffer from AD, and the figure is estimated to triple by 2050.^[1]^ Despite the growing prevalence of AD, only a few treatments have been approved for use by the Food and Drug Administration. These include angiotensin-converting enzyme inhibitors (donepezil, galantamine, and rivastigmine) and N-methyl D-aspartate receptor blockers (memantine).^[2]^ However, these treatments are single-target approaches and provide symptomatic relief rather than disease modification. They are also known to have adverse effects, such as nausea, vomiting, diarrhea, headache, dizziness, fatigue, muscle spasms, and insomnia.^[3]^

Herbal medicine has long been widely used to enhance the treatment of symptoms of dementia and improve cognitive impairment in East Asian countries. A recent study demonstrated that Bojungikgitang (in Korean, Bozhongyiqitang in Chinese, Hochuekkito in Japanese) may inhibit amyloid-β aggregation and increase antioxidant activity and therefore successfully be used in the treatment of AD.^[4]^ Several other clinical studies show that Ukgansan (in Korean, Yigansan in Chinese, Yokukansan in Japanese) improves behavioral and psychological symptoms associated with multiple types of dementia.^[5]^ A retrospective study^[6]^ showed that compared to conventional therapy alone, adding Chinese herbal medicine had significant benefits in AD patients, which were more pronounced with time. In this study, cognitive decline was substantially decelerated in cases of moderate severity, while the cognitive function was largely stabilized in cases of mild severity over 2 years.

Emerging randomized controlled trials (RCTs) also continuously report the effectiveness and safety of herbal medicine for AD. A systematic review and a meta-analysis of these studies were performed to evaluate the efficacy and safety of Chinese herbal medicine to treat cognitive decline in AD,^[3,7,8]^ but no systematic review or meta-analysis has been performed to evaluate the clinical efficacy or safety of using all types of herbal medicine, including Western herbal medicine. Furthermore, existing systematic reviews and meta-analysis are limited to the evaluation of typical cognitive functions, such as Mini-Mental State Examination (MMSE).

The aim of this study are as follows:

1. To assess whether or not herbal medicine is more effective and safer than conventional Western medicine therapies or placebo to treat AD
2. To assess whether or not herbal medicine plus conventional Western medicine is safer and more effective than conventional Western medicine therapies alone to treat AD

## 2 Methods

### 2.1 Study registration

The current protocol report adheres to the preferred reporting items for systematic reviews and meta-analysis (PRISMA) protocols.^[9]^ The protocol for this systematic review and meta-analysis has been registered in Research Registry 2020 under number review registry 933**.**

### 2.2 Eligible criteria for study selection

#### 2.2.1 Types of studies

Only RCTs of herbal medicine treating AD will be included in this study, without publication or language restriction. Non-RCTs, case reports, case series, uncontrolled trials, and experimental studies will be excluded, as well as trials that fail to provide detailed results.

#### 2.2.2 Types of participants

Patients clinically diagnosed with AD will be included, regardless of the age, sex, ethnicity, symptom severity, disease duration, educational background, and clinical setting. Diagnostic and Statistical Manual of Mental Disorders, fourth edition (DSM-IV), National Institute for Neurological and Communicative Disorders and Stroke-Alzheimer's Disease and Related Disorders Association, etc. will be used to make the diagnosis of AD. Patients who have other brain diseases that may affect cognitive function will be excluded, such as stroke, Parkinson disease, traumatic brain injury, etc. Patients will also be excluded if they have a rare form of dementia other than AD, such as Lewy body, frontotemporal, or vascular dementia.

#### 2.2.3 Types of interventions

We will include studies using herbal medicine as the experimental intervention, with no limitations on dosage, frequency, or duration of treatment. We will allow any formulation of herbal medicine (e.g., decoctions, tablets, capsules, pills, and powders); however, we will only include studies in which herbal medicine is administered orally. Intravenous or acupuncture point injections will be excluded. The control intervention will include no treatment, placebo, or conventional medicine. Herbal medicine alone and concurrent treatment with conventional therapy will both be considered acceptable if herbal medicine is applied only to the intervention group and conventional treatment is provided equally to both the intervention and control groups. We will exclude studies comparing different types of herbal medicine and other traditional East Asian medicine therapeutic modalities, such as acupuncture or moxibustions.

#### 2.2.4 Types of outcome measures

For the primary outcome, we will assess the MMSE score to evaluate the cognitive function. For secondary outcomes, we will evaluate the Alzheimer's Disease Assessment Scale-Cognitive Subscale and Montreal Cognitive Assessment test for Dementia scores for further evaluation of the cognitive function. Neuropsychiatric Inventory and Behavioral Pathology in Alzheimer's Disease Rating Scale scores for behavioral and psychological symptoms of dementia and plasma levels of amyloid-beta will also be included as secondary outcomes. We will also look at the number and severity of adverse events.

### 2.3 Search methods for identification of studies

#### 2.3.1 Electronic searches

The following databases will be searched from inception to June 2020: MEDLINE (via PubMed), Cochrane Central Register of Controlled Trials, Excerpta Medica Database, China National Knowledge Infrastructure, Oriental Medicine Advanced Searching Integrated system, Korean Traditional Knowledge Portal, and Citation Information by National Institute of Informatics. Table 1 shows strategy details for PubMed. We will make relative modifications in accordance with the requirements, and an equivalent translation of the search terms will be adopted to ensure that the same searching terms are used in all databases.

**Table 1:**
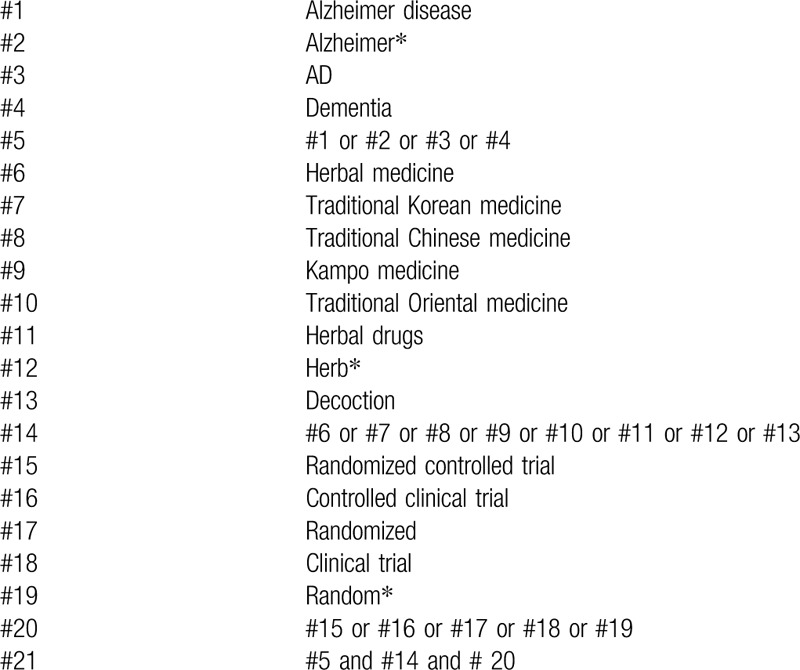
Search strategy for PubMed.

#### 2.3.2 Search for other resources

A manual search will be performed for reference lists of the relevant articles on Google Scholar to identify more studies.

### 2.4 Data collection and analysis

#### 2.4.1 Study selection

**Figure 1:**
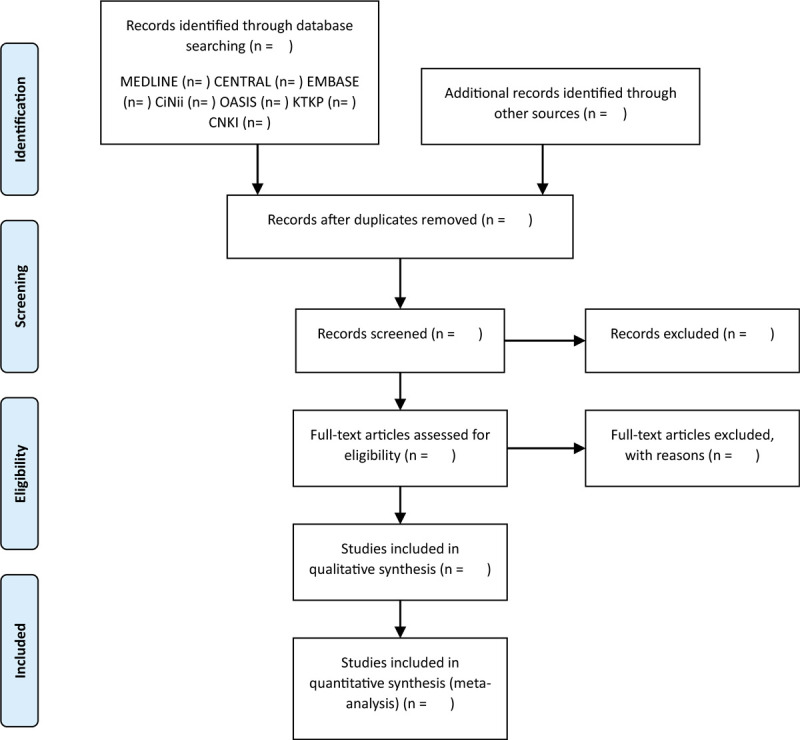
Preferred reporting items for systematic reviews and meta-analysis flow diagram of literature screening and selection processes.

Two review authors (JEL and SK) will independently screen the titles and abstracts to extract the potentially eligible studies according to the inclusion criteria. After removing duplicates, further examination will be subsequently performed by reviewing the full texts. Any diversity between the 2 authors will be resolved by a third (CJ) author through a discussion. The selection will be performed according to the PRISMA flow chart shown in Figure 1. All studies identified by both electronic and manual searches will be uploaded to EndNote X7 (Clarivate Analytics).

#### 2.4.2 Data extraction and management

Two independent review authors (JEL and SK) will extract data according to a predefined data collection form. The form will consist of the general information (such as the authors’ name, title, publication year, country), study methods (such as interventions and comparisons, duration of the intervention and follow-up, study design, sample size, details of randomization, blinding, and any other bias information), participants (characteristics), and outcomes (primary, secondary and side effects). Disagreements will be resolved by a third author (CJ).

#### 2.4.3 Assessment of the bias risk and quality of included studies

The risk of bias in each included study will be conducted by 2 independent review authors (JEL and SK) using the Cochrane Risk-of-Bias Tool. The tool includes random sequence generation, allocation concealment, blinding of participants and personnel, blinding of outcome assessment, incomplete outcome data, selective reporting, and other biases. Each domain will be assessed as a high, unclear, or low risk of bias. Disagreements will be resolved by a third author (CJ).

#### 2.4.4 Measurement of the treatment effect

For continuous data, pooled results will be expressed as the mean difference (MD) or standardized MD with 95% confidence interval (CI). For dichotomous data, pooled results will be expressed as the risk ratio with 95% CI.

#### 2.4.5 Managing missing data

For any missing, insufficient, or unclear data, we will contact the corresponding author to request for adequate information and details of the studies included. If the information cannot be obtained, only the remaining available information will be analyzed and discussed.

#### 2.4.6 Assessment of heterogeneity

We will perform the I^2^ test to evaluate the statistical heterogeneity. If I^2^ is greater than 50%, statistical heterogeneity will be significantly considered. Heterogeneity in the research methodology related to the specific information of intervention, such as types, usages, dose, etc., will also be evaluated.

#### 2.4.7 Data synthesis

The synthesis will be performed using RevMan V.5.3.5 provided by Cochrane Collaboration (V.5.3.5 Copenhagen: The Nordic Cochrane Centre. The Cochrane Collaboration, 2014). If I^2^ ≤ 50%, the fixed-effects model will be employed to evaluate the outcome data. Otherwise, the random-effects model will be applied. If evident heterogeneity is found between studies, a subgroup analysis will be performed to identify the potential reasons for this heterogeneity. Studies will be synthesized according to the type of intervention and/or control as follows:

1. Herbal medicine + conventional western medicine therapy versus conventional Western medicine therapy
2. Herbal medicine + conventional Western medicine therapy versus placebo + conventional Western medicine therapy
3. Herbal medicine versus conventional Western medicine therapy
4. Herbal medicine versus placebo

Heterogeneity levels will be assessed in the included literature, and if enough studies are available to investigate the causes of heterogeneity and its criteria, the groups mentioned below (Analysis of subgroups or subsets section) will be assessed. We will use Grading of Recommendations Assessment, Development and Evaluation pro-software from Cochrane Systematic Reviews to create a Summary of Findings table.

#### 2.4.8 Subgroup analysis

If enough studies are available to investigate the cause of heterogeneity and its criteria, we will conduct a subgroup analysis to detect heterogeneity between groups. It will be performed according to the following aspects:

1. Form of the herbal medicine used, such as granule or decoction
2. Name of the herbal medicine used
3. Sex and age of patients
4. Treatment duration
5. Duration and severity of the disease
6. Type of control, such as no treatment, placebo, or conventional treatment

#### 2.4.9 Sensitivity analysis

We will perform a sensitivity analysis to verify the robustness of the study results. This will be achieved by assessing the impact of the sample size, high risk of bias, missing data, and selected models. Following the analyses, if the quality of a study is judged to be low, it will be removed to ensure the robustness of the results.

#### 2.4.10 Ethics and dissemination

Formal ethical approval is not required in this protocol. We will collect and analyze data based on published studies, and since no patients are directly or specifically assessed in this study, individual privacy will not be a concern. The results of this review will be disseminated to peer-reviewed journals or presented at a relevant conference.

## 3 Discussion

AD adversely affects not only the physical function but also the quality of life, resulting in an extensively increasing burden for both the patients and their caregivers.^[10]^ However, there is yet no disease-modifying treatment, and therefore, effective treatment strategies for AD are required.

Studies have shown that herbal medicine has the potential to treat the symptoms of AD, and this might be due to the multitarget intervention effects of herbal medicine. The possible mechanisms for this are thought to be anti-inflammatory, antiapoptotic, and antioxidant actions,^[11]^ which include protecting neurons from amyloid-β damage, inhibiting amyloid-β secretion, and suppressing amyloid-β-induced oxidative stress and apoptosis.^[12]^ In this regard, herbal medicine may have the potential to overcome the limitations of conventional medicine.

The present review will be conducted to assess the effectiveness and safety of herbal medicine in AD, aimed at establishing management strategies to benefit and lessen the burden of practitioners as well as patients and their families.

## Acknowledgments

This study was supported by the Traditional Korean Medicine R&D program funded by the Ministry of Health & Welfare through the Korea Health Industry Development Institute (KHIDI, HB16C0051).

## Author contributions

**Conceptualization:** JiEun Lee, Seungwon Kwon

**Data curation:** Chul Jin, Seung-Yeon Cho, Woo-Sang Jung

**Formal analysis:** Chul Jin, Sang-Kwan Moon, Seong-Uk Park, Jung-Mi Park

**Funding acquisition:** Ki-Ho Cho

**Project administration:** Seungwon Kwon

**Writing – original draft:** JiEun Lee, Seungwon Kwon

**Writing – review & editing:** Seungwon Kwon, Ki-Ho Cho, Chang-Nam Ko
